# Correction: Increased levels of soluble triggering receptor expressed on myeloid cells sTREM1 in ICU patients with cardiovascular disease and associated organ dysfunction

**DOI:** 10.1186/cc11143

**Published:** 2011-12-30

**Authors:** S Dewan, A Varma, N Verma, M Talegaonkar

**Affiliations:** 1 Fortis Escorts Heart Institute, New Delhi, India

## Correction

After publication of this abstract [1], we found that not all authors were listed. A full author list is provided here. We regret any inconvenience this error may have caused.
